# Novel Role for Protein Inhibitor of Activated STAT 4 (PIAS4) in the Restriction of Herpes Simplex Virus 1 by the Cellular Intrinsic Antiviral Immune Response

**DOI:** 10.1128/JVI.03055-15

**Published:** 2016-04-14

**Authors:** Kristen L. Conn, Peter Wasson, Steven McFarlane, Lily Tong, James R. Brown, Kyle G. Grant, Patricia Domingues, Chris Boutell

**Affiliations:** MRC-University of Glasgow Centre for Virus Research (CVR), Glasgow, Scotland, United Kingdom

## Abstract

Small ubiquitin-like modifier (SUMO) is used by the intrinsic antiviral immune response to restrict viral pathogens, such as herpes simplex virus 1 (HSV-1). Despite characterization of the host factors that rely on SUMOylation to exert their antiviral effects, the enzymes that mediate these SUMOylation events remain to be defined. We show that unconjugated SUMO levels are largely maintained throughout infection regardless of the presence of ICP0, the HSV-1 SUMO-targeted ubiquitin ligase. Moreover, in the absence of ICP0, high-molecular-weight SUMO-conjugated proteins do not accumulate if HSV-1 DNA does not replicate. These data highlight the continued importance for SUMO signaling throughout infection. We show that the SUMO ligase protein inhibitor of activated STAT 4 (PIAS4) is upregulated during HSV-1 infection and localizes to nuclear domains that contain viral DNA. PIAS4 is recruited to sites associated with HSV-1 genome entry through SUMO interaction motif (SIM)-dependent mechanisms that are destabilized by ICP0. In contrast, PIAS4 accumulates in replication compartments through SIM-independent mechanisms irrespective of ICP0 expression. Depletion of PIAS4 enhances the replication of ICP0-null mutant HSV-1, which is susceptible to restriction by the intrinsic antiviral immune response. The mechanisms of PIAS4-mediated restriction are synergistic with the restriction mechanisms of a characterized intrinsic antiviral factor, promyelocytic leukemia protein, and are antagonized by ICP0. We provide the first evidence that PIAS4 is an intrinsic antiviral factor. This novel role for PIAS4 in intrinsic antiviral immunity contrasts with the known roles of PIAS proteins as suppressors of innate immunity.

**IMPORTANCE** Posttranslational modifications with small ubiquitin-like modifier (SUMO) proteins regulate multiple aspects of host immunity and viral replication. The protein inhibitor of activated STAT (PIAS) family of SUMO ligases is predominantly associated with the suppression of innate immune signaling. We now identify a unique and contrasting role for PIAS proteins as positive regulators of the intrinsic antiviral immune response to herpes simplex virus 1 (HSV-1) infection. We show that PIAS4 relocalizes to nuclear domains that contain viral DNA throughout infection. Depletion of PIAS4, either alone or in combination with the intrinsic antiviral factor promyelocytic leukemia protein, significantly impairs the intrinsic antiviral immune response to HSV-1 infection. Our data reveal a novel and dynamic role for PIAS4 in the cellular-mediated restriction of herpesviruses and establish a new functional role for the PIAS family of SUMO ligases in the intrinsic antiviral immune response to DNA virus infection.

## INTRODUCTION

Intrinsic antiviral immunity is the first line of intracellular defense to viral infection. This defense is mediated by constitutively expressed cellular proteins that cooperatively act to restrict the progression of infection (reviewed in references 1 to 4). However, viruses have evolved mechanisms to counteract this host response to ensure their efficient replication and spread.

During herpes simplex virus 1 (HSV-1) infection, viral gene expression occurs in a tightly regulated temporal cascade consisting of immediate early (IE), early (E), and late (L) gene products. IE proteins play pivotal roles in modulating the intracellular environment in order to facilitate viral replication, including inactivation of host immunity that would otherwise restrict the progression of infection (reviewed in reference 5). One key aspect of intrinsic immunity to HSV-1 infection is the rapid recruitment of constitutively expressed restriction factors to nuclear sites associated with viral genomes following their nuclear entry (6–8). Left unimpeded, the stable recruitment of such factors is sufficient to restrict HSV-1 gene expression and facilitate the transcriptional silencing of viral genomes to block lytic replication (6, 7, 9–13). Restriction factors recruited to infecting viral genomes include core constituent proteins of promyelocytic leukemia (PML) nuclear bodies (PML-NBs; also known as nuclear domain 10 [ND10]), proteins involved in the DNA damage response (DDR), and the nuclear DNA pathogen sensor IFI16 (6, 7, 11, 12, 14–16). The small ubiquitin-like modifier (SUMO) pathway (17–19) mediates the recruitment of PML-NB-associated restriction factors, although to what extent this pathway is involved in the recruitment of other restriction factors remains unknown.

The posttranslational modification (PTM) of proteins with SUMO regulates numerous cellular processes, including multiple aspects associated with virus infection and host immunity (reviewed in references 3, 20, and 21). Three predominant SUMO isoforms (SUMO1 to -3) are ubiquitously expressed and conjugated within mammalian cells. SUMOylation is analogous to ubiquitination, requiring an E1 activating complex (SAE1/2), an E2 conjugation enzyme (Ubc9), and E3 ligases that impart substrate specificity (reviewed in references 22 and 23). SUMO2 and SUMO3 (here referred to as SUMO2/3) share 97% amino acid identity and can form polymeric SUMO chains (24). SUMO1 shares 46% amino acid identity with SUMO2 and is typically associated with single modification or the termination of poly-SUMO chains (24). Proteins can be deSUMOylated by SUMO-specific proteases (reviewed in references 25 and 26). SUMOylation therefore provides a highly dynamic mechanism that enables the cell to quickly respond to various stimuli by altering protein stability, functionality, or intracellular localization.

Following nuclear entry of HSV-1 genomes, *de novo* SUMO conjugates accumulate adjacent to viral genomes in a Ubc9-dependent manner (18). This stimulates the recruitment of constituent PML-NB restriction factors, including PML, Sp100, and Daxx, through SUMO-dependent protein-protein interactions mediated by their respective SUMO interaction motifs (SIMs) (17, 18). Importantly, the recruitment of these host factors occurs independently of PML and is inhibited by Ubc9 depletion, highlighting a key role for the SUMO pathway in this aspect of intrinsic immunity (18). The HSV-1 IE protein ICP0, a viral SUMO-targeted ubiquitin ligase (STUbL), counteracts this host response by inducing the degradation of PML-NB constituent, and other SUMO-conjugated, proteins through a combination of SUMO-dependent and -independent targeting mechanisms (3, 18, 19, 27–30). ICP0-mediated proteasome-dependent degradation or dispersal of restriction factors away from infecting viral genomes enables the progression of viral gene expression and the onset of lytic replication (reviewed in reference 31). HSV-1 mutants that lack, or express catalytically inactive, ICP0 are therefore more susceptible to cellular restriction and are likely to enter into a nonproductive (quiescent) infection. A high multiplicity of infection (MOI) with ICP0-null mutant HSV-1 is sufficient, however, to saturate and overcome this host response (reviewed in reference 31). Under such conditions, the onset of infection results in a global induction of high-molecular-weight (HMW) SUMO-conjugated proteins (18, 19). However, the SUMO ligases responsible for regulating these nuclear responses to infection remain to be defined.

In contrast to the ubiquitin pathway, the SUMOylation of cellular proteins is regulated by a relatively small number of E3 ligases, the best-characterized and largest family of which is the protein inhibitor of activated STAT (PIAS1 to -4) family. These ligases were initially characterized as negative regulators of innate immunity by virtue of their transrepression of key regulators in the interferon (IFN) pathway (32–37). However, members of this family have subsequently been shown to play important roles in the regulation of many other cellular processes, including PML stability (38), transcription (39), DNA repair (40–42), NF-κB regulation (43, 44), the cell cycle (45, 46), and senescence (47). PIAS family members contain several evolutionarily conserved domains and motifs that mediate nuclear matrix binding (SAP), noncovalent SUMO interaction (SIM), nuclear retention (PINIT), transrepression activity (LxxLL), and SUMO ligase activity (Siz PIAS [SP] RING) (reviewed in references 48 and 49). Due to the varied nature of nuclear substrates and cellular processes that PIAS proteins are reported to regulate, we investigated if members of this family mediated aspects relating to intrinsic antiviral immunity to HSV-1 infection. We report that PIAS4 localized to sites associated with viral DNA throughout infection. Following nuclear entry of viral genomes, PIAS4 relocalized to the nuclear periphery in a SIM-dependent manner. The stable association of PIAS4 at foci associated with infecting viral genomes was inhibited by ICP0. PIAS4 also accumulated in viral replication compartments, irrespective of ICP0 expression, in a SAP domain- and LxxLL motif-dependent manner. Depletion of PIAS4 did not affect the replication of wild-type HSV-1 but significantly enhanced the replication of ICP0-null mutant HSV-1. Codepletion of PIAS4 and PML enhanced the relief in cellular restriction, indicating that PML and PIAS4 independently contribute to the restriction of ICP0-null mutant HSV-1. We therefore identify a unique role for PIAS4 as a positive regulator of intrinsic antiviral immunity to HSV-1 infection and uncover a previously unknown role for this family of SUMO ligases in the cellular restriction of DNA viruses.

## MATERIALS AND METHODS

### Cells, drugs, and viruses.

Human osteosarcoma (U2OS) cells, human embryonic kidney (HEK-293T) cells, and primary human foreskin fibroblasts (HFs) (Department of Urology, University of Erlangen [50]) were maintained at 37°C in 5% CO_2_ in Dulbecco's modified Eagle medium (DMEM; Life Technologies; 41966) supplemented with 10% fetal bovine serum (FBS; Life Technologies; 10270), 100 U/ml of penicillin, and 100 μg/ml of streptomycin (Life Technologies; 15140-122). HFt cells are immortalized human foreskin fibroblasts stably transduced with retrovirus that expresses the catalytic subunit of human telomerase, as described previously (51). The plaque-forming efficiency of wild-type or ICP0-null mutant HSV-1 strains in HFt cells is similar to that in HFs cells (data not shown).

Phosphonoacetic acid (PAA; Sigma-Aldrich; 284270) prepared at 100 mg/ml in DMEM was used at 400 μg/ml. Acycloguanosine (ACG; Sigma-Aldrich; A4669) prepared at 50 mM in Milli-Q H_2_O was used at 50 μM. MG132 (Calbiochem; 474790) prepared at 10 mM in dimethyl sulfoxide (DMSO; Sigma-Aldrich; D2650) was used at 10 μM. Doxycycline (DOX; Sigma-Aldrich; D9891) prepared at 1 mg/ml in Milli-Q H_2_O was used at 0.1 μg/ml. Cycloheximide (CHX; Sigma-Aldrich; C-7698) prepared at 5 mg/ml in Milli-Q H_2_O was used at 10 μg/ml. For transgenic cells, hygromycin (Invitrogen; 10687-010), puromycin (Puro; Sigma-Aldrich; P8833), or neomycin (Neo; Sigma-Aldrich; A1720) was used at 50 μg/ml, 1 μg/ml, or 1 mg/ml, respectively, during selection or 5 μg/ml, 0.5 μg/ml, or 0.5 mg/ml, respectively, for maintenance.

Wild-type HSV-1 strain 17*syn*+, its ICP0-null mutant derivative *dl*1403 (52), and their respective variants that express eYFP.ICP4 (53) were used. All viruses were propagated and titrated as described previously (54). When used, MG132, PAA, or ACG was added following a 1-h inoculum adsorption.

### Plasmids and lentiviral transduction.

The FLAG sequence in FLAG.PIAS4, a generous gift from Mary Dasso (NIH, Bethedsa, MD), was replaced with an enhanced yellow fluorescent protein (eYFP) coding sequence, and the resultant eYFP.PIAS4 expression construct was subcloned into the lentiviral vector pLKO.dCMV.TetO/R (55) via NheI and SalI restriction sites. PIAS4 mutations and deletions performed by PCR mutagenesis are described in Table 1. All clones were confirmed by sequencing. His-tagged SUMO2, a generous gift from Ben Hale (University of Zurich), was subcloned into the lentiviral vector pLVX.neo (Invitrogen) via Xhol and Xbal restriction sites. Plasmids that express short hairpin RNAs (shRNAs) against PML (shPML) or a control sequence (shCtrl) are described elsewhere (18, 50). Of the five tested shRNAs against PIAS4 (Sigma-Aldrich), shP4_3 (based on 5′-AATTCGGTGGGCTTCAGCA-3′) had the best levels of depletion and was selected for use.

**TABLE 1:** PCR primers used for the mutation or deletion of conserved domains within PIAS4

PIAS4 mutant	Forward primer	Reverse primer	Amino acid sequence change
C342A	5′-TGCCGGGCAGAGACCGCCGCCCACCTGCAGT	5′-ACTGCAGGTGGGCGGCGGTCTCTGCCCGGCA	C to A
mLxxLL	5′-TCAGATGGCCGCGGGTTTCGTGGGCCGGAGTA	5′-CCGCGGCCATCTGAAGGTCGGAGACT	LQMLL to LQMAA
ΔSAP (N)	5′-TAACGCTAGCACCATGGTGAGC	5′-GGCTCCGAGTTCTTCTTGGCCATCCGGGAATTCGAAGCTT	Deletion encompassing A2 to A67
ΔSAP (C)	5′-GCCAAGAAGAACTCGGAGCC	5′-TTAGTCGACCCGTCAGCAGGCCGGCACCA	
mPINIT	5′-CGCCCCGCCAACGCCACTCAC	5′-GTGAGTGGCGTTGGCGGGGCG	PINIT to PANAT
mSIM	5′-GATGCGGCGGACCTCACGCTGGACAGCTC	5′-GACCTCCGCCGCATCGGCGCCCGGCTTCC	VVDLT to AADLT
ΔC34	5′-TTTTTGCTAGCACCATGGTGAGCAAG	5′-TTTTGTCGACTCACGAGGACGATGA	Stop codon introduced after S476

Lentiviral supernatant stocks were generated and HFt cells were transduced as described previously (50). Pooled stably transduced cells were used for experimentation.

### Antibodies.

Primary rabbit antibodies included anti-actin (Sigma-Aldrich; A5060), anti-enhanced green fluorescent protein (anti-eGFP; Abcam; ab290), anti-Daxx (Upstate; 07-471), anti-PIAS1 (LsBio; LS-B9173), anti-PIAS2 (LsBio; LS-C108717), anti-PIAS3 (LsBio; LS-C98795), anti-PIAS4 (LsBio; LS-C108719), anti-PML (Bethyl Laboratories; A301-167A), anti-Sp100 (SpGH [56]), anti-SUMO1 (Abcam; ab32058), and anti-SUMO2/3 (Abcam; ab22654) antibodies. Primary mouse monoclonal antibodies included anti-His (Abcam; ab18184), anti-ICP0 (11060 [57]), anti-ICP4 (58s [58]), anti-SUMO1 (Invitrogen; 33-2400), anti-SUMO2/3 (Abcam; ab81371), anti-UL42 (Z1F11 [59]), and anti-VP5 (DM165 [60]) antibodies. Secondary antibodies included DyLight 680- or 800-conjugated anti-rabbit or -mouse (Thermo) antibodies; Alexa 488-, 555-, or 633-conjugated anti-rabbit, -sheep, or -mouse (Invitrogen) antibodies; and peroxidase-conjugated anti-mouse antibodies (Sigma-Aldrich; A4416).

### Plaque forming efficiency (PFE).

HFt cells (1 × 10^5^ per well) in 24-well plates were incubated at 37°C in 5% CO_2_ overnight or at least 4 h prior to infection. Wild-type or ICP0-null mutant HSV-1 was serially diluted in serum-free DMEM. Cells inoculated with sequential viral dilutions were rocked and rotated every 10 min for 1 h and then overlaid with 37°C cell appropriate medium supplemented with 2% human serum (HS). At 24 to 26 h postinfection (hpi), cells were washed twice in phosphate-buffered saline (PBS; Sigma-Aldrich; D1408), fixed in 1.8% formaldehyde (Sigma-Aldrich; F8775) and 0.1% NP-40 (BDH; 56009) in PBS for 10 min, and then washed twice in 0.1% Tween in PBS (PBST). Cells were blocked with 5% skim milk powder (SMP; Marvel) in PBST for 30 min before incubation with anti-VP5 diluted in 5% SMP in PBST for 90 min. Cells were washed in PBST three times, incubated with peroxidase-conjugated anti-mouse IgG diluted in 5% SMP in PBST for 60 min, and then washed in PBST three more times. Plaques were visualized with True Blue peroxidase developing solution (Insight; 50-78-02) according to the manufacturer's instructions.

### Western blotting.

HFt cells were washed twice with room temperature PBS and then lysed and collected in SDS-PAGE loading buffer with 4 M urea (Sigma-Aldrich; U0631) and 50 mM dithiothreitol (DTT; Sigma-Aldrich; D0632). Whole-cell lysates were immediately used or snap-frozen. Proteins resolved using standard Tris-glycine or Tris-tricine SDS-PAGE systems were electrotransferred onto 0.2-μm nitrocellulose membranes (Amersham; 15249794) for 150 (Tris-tricine) or 180 (Tris-glycine) min at 250 mA in Towbin buffer (25 mM Tris, 192 mM glycine, 20% [vol/vol] methanol) at room temperature. Membranes were blocked in 5% newborn calf serum (NCS) in PBS at 4°C overnight. Subsequent steps were performed at room temperature on a roller apparatus. Membranes were incubated in primary antibody diluted in 5% NCS in PBST for 2 h, washed in PBST three times for 5 min each, then incubated in secondary antibody diluted 1:10,000 in 5% NCS in PBST for 1 h. Following three 5-min washes in PBST, one 5-min wash in PBS, and one rinse in Milli-Q H_2_O, membranes were imaged on an Odyssey infrared imager (LiCor). The intensity of protein bands was quantified with Odyssey Image Studio software.

### Immunofluorescence and confocal microscopy.

HFt cells (1 × 10^5^) seeded on 13-mm glass coverslips in 24-well plates were incubated at 37°C in 5% CO_2_ overnight. For infections, wild-type or ICP0-null mutant HSV-1 was diluted in serum-free DMEM to the multiplicity of infection (MOI) indicated in the corresponding figure legends. Cells were typically infected at a low MOI for 16 to 24 h in order to examine the recruitment of host factors to nuclear sites associated with HSV-1 genome entry or replication compartments in cells at the periphery of a developing plaque (6). Inoculum was adsorbed for 1 h at 37°C in 5% CO_2_, with rocking and rotating every 10 min, prior to the addition of 37°C cell-appropriate medium with 2% HS. Fixation and immunostaining were performed at room temperature. Cells were washed twice in CSK (10 mM HEPES, 100 mM NaCl, 300 mM sucrose, 3 mM MgCl_2_, 5 mM EGTA), fixed and permeabilized in 1.8% formaldehyde and 0.5% Triton X-100 (Sigma-Aldrich; T-9284) in CSK for 10 min, and then washed in CSK three times. Coverslips were blocked with 2% HS in PBS for 10 min, incubated with primary antibodies diluted in 2% HS in PBS for 90 min, and then washed in PBS three times. Cells were then incubated with 4′,6-diamidino-2-phenylindole (DAPI; Sigma-Aldrich; D9542) and secondary antibodies diluted 1:1,000 in 2% HS in PBS for 60 min, washed in PBS three times, and washed twice in Milli-Q H_2_O. Coverslips were mounted on glass slides using a glycerol-based mounting medium (Citifluor; AF1) sealed with nail enamel. Cells were examined using Zeiss LSM 510 or LSM 710 confocal microscopes with 405-nm, 488-nm, 543-nm, and 633-nm laser lines under a 63× Plan-Apochromat oil immersion lens with a numerical aperture of 1.4. For graphing purposes, pixel intensities were acquired using Zen software (Zeiss). Exported images were processed with minimal adjustment using Adobe Photoshop and assembled for presentation using Adobe Illustrator.

### Quantitative reverse transcription-PCR (RT-PCR).

A total of 1.5 × 10^5^ or 2 × 10^5^ HFt cells seeded in 12-well plates were incubated at 37°C in 5% CO_2_ overnight or at least 4 h before further manipulation. Total RNA was isolated using the RNAeasy Plus kit (Qiagen; 74134) or TRIzol reagent (Invitrogen; 15596) according to the manufacturer's instructions. RNA was reverse transcribed using the TaqMan reverse transcription reagent kit (Life Technologies; N8080234) with oligo(dT) primers. Samples were analyzed in triplicate using TaqMan Fast Universal PCR master mix (Life Technologies; 4352042) with the following TaqMan gene-specific primer (6-carboxyfluorescein [FAM]/MGB)-probe mixes (Life Technologies): PML (assay ID Hs00231241_m1), PIAS4 (assay ID Hs00249203_m1), 18S (GenBank accession number X03205.1; Thermo Fisher Scientific; 4319413E), or glyceraldehyde-3-phosphate dehydrogenase (GAPDH; GenBank accession number NM_002046.5; Thermo Fisher Scientific; 4333764F).

## RESULTS

### HMW SUMO-conjugated protein levels increase when HSV-1 DNA replicates.

**FIG 1:**
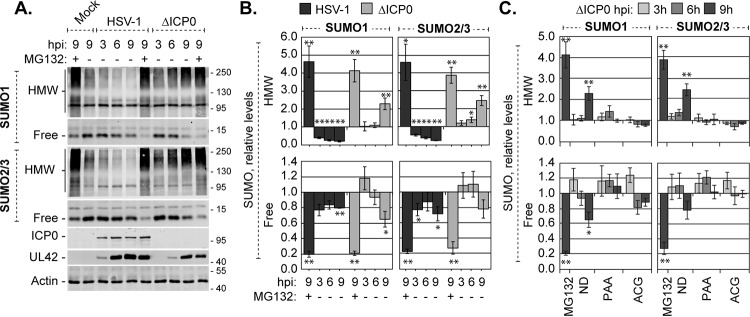
HMW SUMO-conjugated proteins do not accumulate when HSV-1 DNA does not replicate. (A) Western blots show the levels of HMW-conjugated or free SUMO1 or -2/3 during wild-type or ICP0-null mutant HSV-1 infection. HFt cells infected with 10 PFU of wild-type or ICP0-null mutant (ΔICP0) HSV-1 per cell in the presence (+) or absence (−) of the proteasome inhibitor MG132 were harvested at 3, 6, or 9 h postinfection (hpi). Membranes were probed for SUMO1 or -2/3, the viral protein ICP0 or UL42 to monitor infection progression, or actin as a loading control. Molecular mass markers are indicated, in kilodaltons. (B) Bar graphs show the average relative levels of HMW-conjugated or free SUMO1 or -2/3 during wild-type or ICP0-null mutant HSV-1 infection. The intensities of SUMO1 or -2/3 protein bands were quantitated from Western blots as shown in panel A; quantitated levels were normalized to their respective loading controls and are expressed relative to the levels in mock-infected cells at 9 hpi (1.0). Means and standard error of the means (SEM) are shown (*n* ≥ 4). (C) Bar graphs show the average relative levels of HMW-conjugated or free SUMO1 or -2/3 in the presence of either of the DNA replication inhibitors PAA or ACG, MG132, or no drug (ND). Infections equivalent to those for panel A were performed in the presence or absence of the indicated drugs. The intensities of SUMO1 or -2/3 protein bands were quantitated from Western blots; quantitated values were normalized to their respective loading controls and are expressed relative to the levels in untreated mock-infected cells at 9 hpi. Means and SEM are shown (*n* ≥ 3). *, *P* < 0.05; **, *P* < 0.01 (Student's two-tailed *t* test).

Whether or not the steady-state levels of unconjugated free SUMO were altered during HSV-1 infection along with the global changes in SUMOylation was evaluated. Consistent with previous reports (18), HMW SUMO1- or SUMO2/3-conjugated protein levels decreased during wild-type HSV-1 infection, while they increased during ICP0-null mutant HSV-1 infection (Fig. 1A and B, HMW). Although the levels of HMW SUMO-conjugated proteins were clearly altered in an ICP0-dependent manner, the levels of unconjugated free SUMO1 or -2/3 remained relatively stable (Fig. 1A and B, free). In HFt cells infected with 10 PFU per cell of wild-type or ICP0-null mutant HSV-1, the cellular pools of free SUMO1 or -2/3 were depleted by 20 to 30% at 9 hpi (Fig. 1A and B, free). In contrast, inhibition of the proteasome, in either the presence or absence of ICP0, was sufficient to deplete the pools of free SUMO1 or -2/3 by 70 to 80% (Fig. 1A and B, +MG132). Together, these data indicate that free SUMO levels are likely regulated during infection, irrespective of the broad depletion or accumulation of HMW SUMO-conjugated proteins during wild-type or ICP0-null mutant HSV-1 infection, respectively. Moreover, these data suggest that free SUMO levels are unlikely to be a limiting factor for *de novo* SUMOylation, supporting the concept that dynamic SUMO modification and signaling occur throughout infection (28).

In the absence of ICP0, the accumulation of HMW SUMO-conjugated proteins was most prominent at later times after infection (Fig. 1A and B, 9 hpi). To evaluate whether HSV-1 DNA replication or late (L) protein expression contributed to this accumulation, HFt cells were infected with 10 PFU per cell of ICP0-null mutant HSV-1 in the presence or absence of phosphonoacetic acid (PAA) to inhibit the HSV-1 DNA polymerase, or acycloguanosine (ACG) to inhibit nascent DNA synthesis (61–64). In the absence of viral DNA replication, the levels of HMW SUMO1- or SUMO2/3-conjugated proteins, as well as the levels of free SUMO1 or SUMO2/3, remained similar to those in mock-infected cells (Fig. 1C). The SUMOylation events that result in the accumulation of HMW SUMO-conjugated proteins therefore occur when HSV-1 DNA replicates or L proteins are expressed. Viral transcription in general, or the expression of IE or E proteins, was not sufficient to stimulate this broad accumulation of HMW SUMO-conjugated proteins during ICP0-null mutant HSV-1 infection.

### PIAS4 localizes to HSV-1 replication compartments.

**FIG 2:**
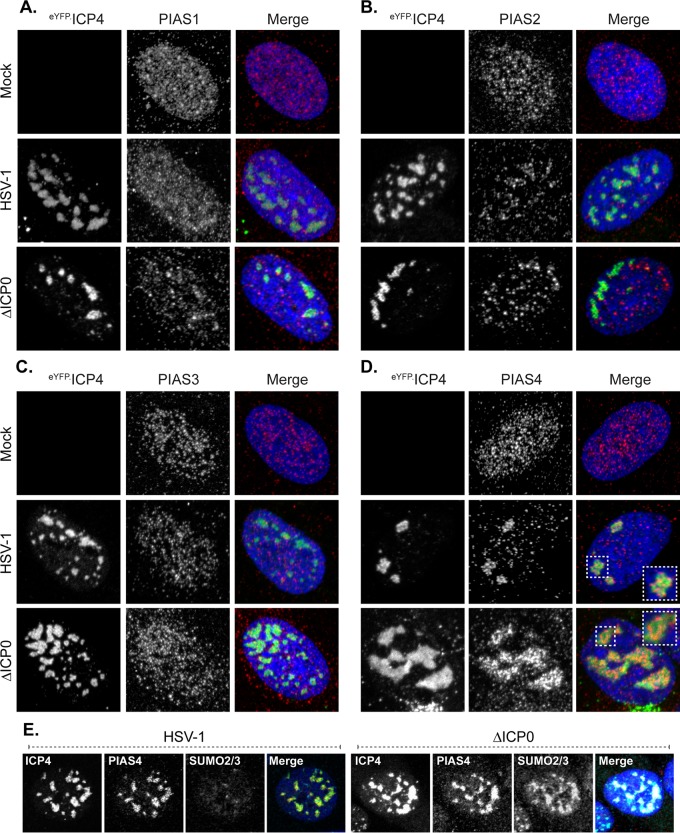
PIAS4 accumulates in HSV-1 replication compartments. (A to D) Confocal images show the nuclear localization of PIAS1 (A), PIAS2 (B), PIAS3 (C), or PIAS4 (D) in HFt cells mock infected (mock) or infected with 0.002 PFU of wild-type HSV-1 or 2 PFU of ICP0-null mutant (ΔICP0) HSV-1 per cell for 16 h. Wild-type and mutant HSV-1 strains expressed eYFP-ICP4, the accumulation of which was used to visualize replication compartments within cells at the edge of developing plaques (6). PIAS proteins were visualized by indirect immunofluorescence (red). Insets (dashed boxes in panel D) highlight regions of PIAS4 localization within replication compartments. (E) Confocal images show the nuclear localization of PIAS4 and SUMO2/3 within wild-type or ICP0-null mutant HSV-1 replication compartments in cells infected as described above. ICP4 (green), PIAS4 (red), and SUMO2/3 (cyan) were visualized by indirect immunofluorescence. Nuclei were visualized by DAPI (blue).

To investigate the potential significance of the accumulation of HMW SUMO-conjugated proteins during ICP0-null mutant HSV-1 infection, the SUMO E3 ligase(s) that could mediate these SUMOylation events was analyzed, starting with the PIAS protein family. Consistent with other reports, PIAS proteins in HFt cells were predominantly nuclear, with microspeckled distributions characteristic of localization at matrix-associated regions (MARs) (Fig. 2A to D, mock) (65–67). While a subpopulation of PIAS1 localized to PML-NBs, substantial localization of PIAS2, PIAS3, or PIAS4 at these structures was not detected in this cell type (Fig. 2A to D, mock, and data not shown) (38, 66). PIAS4 robustly relocalized to viral replication compartments, whereas PIAS1, PIAS2, and PIAS3 did not (Fig. 2A to D). The relocalization of PIAS4 was independent of ICP0, as it occurred during wild-type or ICP0-null mutant HSV-1 infection (Fig. 2D). PIAS4 is thus identified as a SUMO E3 ligase that accumulates in herpesvirus replication compartments. SUMO2/3 also accumulated in HSV-1 replication compartments, although only in the absence of ICP0 (Fig. 2E). Therefore, during ICP0-null mutant HSV-1 infection, the majority of nuclear PIAS4 and SUMO2/3 localize in replication compartments.

### Multiple domains mediate PIAS4 localization at MARs.

**FIG 3:**
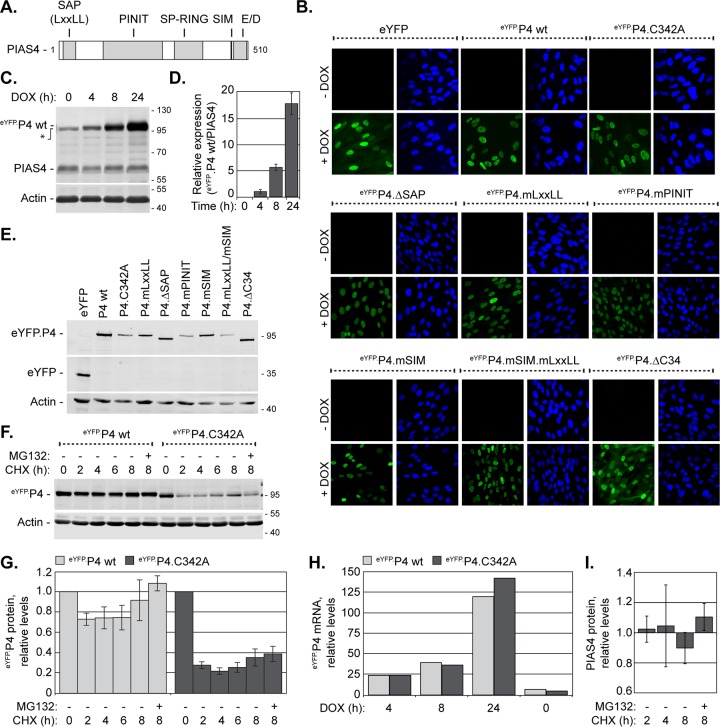
HFt cells stably transduced with DOX-inducible eYFP.PIAS4 wild-type or mutant protein expression constructs. (A) The illustration depicts the conserved PIAS4 domains (gray boxes) mutated within eYFP.PIAS4 (eYFP.P4) (Table 1). (B) Confocal images show the nuclear expression of eYFP, eYFP.P4 wild-type (wt) or mutant proteins (as indicated) in transgenic HFt cells after 8 h of induction (+DOX) or not (−DOX). Nuclei were visualized by DAPI (blue). (C) Western blot shows the levels of eYFP.PIAS4 wild-type (eYFP.P4 wt) or endogenous PIAS4 (PIAS4) in transgenic HFt cells after different lengths of DOX induction, as indicated. Membranes were probed for PIAS4 or actin as a loading control. The asterisk indicates a PIAS4 polyclonal antibody cross-reacting band. (D) A bar graph shows the average level of eYFP.P4 wt relative to endogenous PIAS4 in transgenic cells after various lengths of DOX induction. The intensities of PIAS4 protein bands from Western blots as in panel C were quantitated and normalized to their respective loading controls. Normalized levels of eYFP.P4 wt are expressed relative to the normalized levels of endogenous PIAS4 at each time. Means and standard deviations (SD) are shown (*n* = 3). (E) Whole-cell lysates collected from transgenic HFt cells 8 h post-DOX induction were analyzed by Western blotting for the levels of eYFP and eYFP.PIAS4 wild-type or mutant protein (as indicated) expression. Membranes were probed for eYFP or actin as a loading control. (F) Western blot shows the levels of eYFP.PIAS4 wild-type or catalytically inactive mutant (C342A) proteins at different times after translational inhibition. Expression of eYFP.P4 wt or eYFP.P4.C342A was DOX induced for 4 h prior to the replacement of DOX with the translation inhibitor cycloheximide (CHX) in the presence (+) or absence (−) of MG132. Whole-cell lysates were harvested at 0, 2, 4, 6, or 8 h following CHX addition. Membranes were probed with eYFP or actin as a loading control. Molecular mass markers are indicated, in kilodaltons. (G) A bar graph shows the average relative levels of eYFP.PIAS4 wild-type or catalytically inactive mutant proteins following translation inhibition. The intensities of protein bands corresponding to eYFP.P4 quantitated from Western blots as in panel F were normalized to their respective loading controls and expressed relative to the normalized levels at 0 h of CHX treatment (1.0). Means and SEM are shown (*n* = 3). (H) A bar graph shows the average relative levels of PIAS4 mRNA in transgenic cells that express eYFP.P4 wild-type or catalytically inactive mutant proteins. Total RNA was isolated after 4, 8, or 24 h of DOX induction. The levels of PIAS4 mRNA were determined using the TaqMan system of quantitative RT-PCR. Values were normalized to GAPDH expression using the threshold cycle (ΔΔ*C_T_*) method and are expressed relative to the normalized level of PIAS4 mRNA at 0 h of DOX induction (1). Values represent the averages from two independent experiments. (I) A bar graph shows the average relative level of endogenous PIAS4 following translation inhibition. HFt cells were treated with CHX in the presence (+) or absence (−) of MG132, and whole-cell lysates were collected at 2, 4, or 8 h after CHX addition. Intensities of the protein bands corresponding to PIAS4 were quantitated and normalized to their respective loading controls and are expressed relative to the normalized level of PIAS4 at 0 h of CHX treatment (1.0). Means and SEM are shown (*n* ≥ 3).

To investigate the roles of PIAS4 during HSV-1 infection, the domains that mediate recruitment into replication compartments were evaluated (Fig. 3A). While the SAP domain regulates MAR association (65, 66), the LxxLL sequence, PINIT domain, SIM, or carboxyl (C)-terminal acidic domain (AD) may mediate important protein-protein interactions that regulate PIAS4 localization during infection (Fig. 3A). Additionally, the catalytic activity of PIAS4 may regulate localization through the SUMOylation of substrates to influence relevant protein-protein interactions.

A panel of lentiviral vectors that encode eYFP fused to PIAS4 with point mutations or deletions in the aforementioned domains was constructed (Table 1; Fig. 3A). To regulate expression of the eYFP.PIAS4 wild-type or mutant proteins, transcriptional control was placed under the control of a doxycycline (DOX)-inducible promoter. HFt cells stably transduced with the eYFP.PIAS4 expression constructs did not have detectable eYFP expression in the absence of DOX, whereas greater than 90% of transduced cells had detectable eYFP fluorescence following DOX induction (Fig. 3B). As expected, the relative levels of eYFP fusion protein expression increased with induction time (Fig. 3C and D). The ectopic PIAS4 proteins were of the expected molecular weight; however, several mutant proteins had lower expression levels than the wild-type eYFP.PIAS4 (Fig. 3E). At least for the catalytically inactive mutant, eYFP.P4.C342A, lower expression levels resulted from decreased protein abundance rather than reduced transcript expression (Fig. 3F to H). Endogenous PIAS4 and eYFP.PIAS4 were more stable than eYFP.P4.C342A, suggesting that the catalytically inactive mutant protein was likely inherently unstable (Fig. 3F and I).

**FIG 4:**
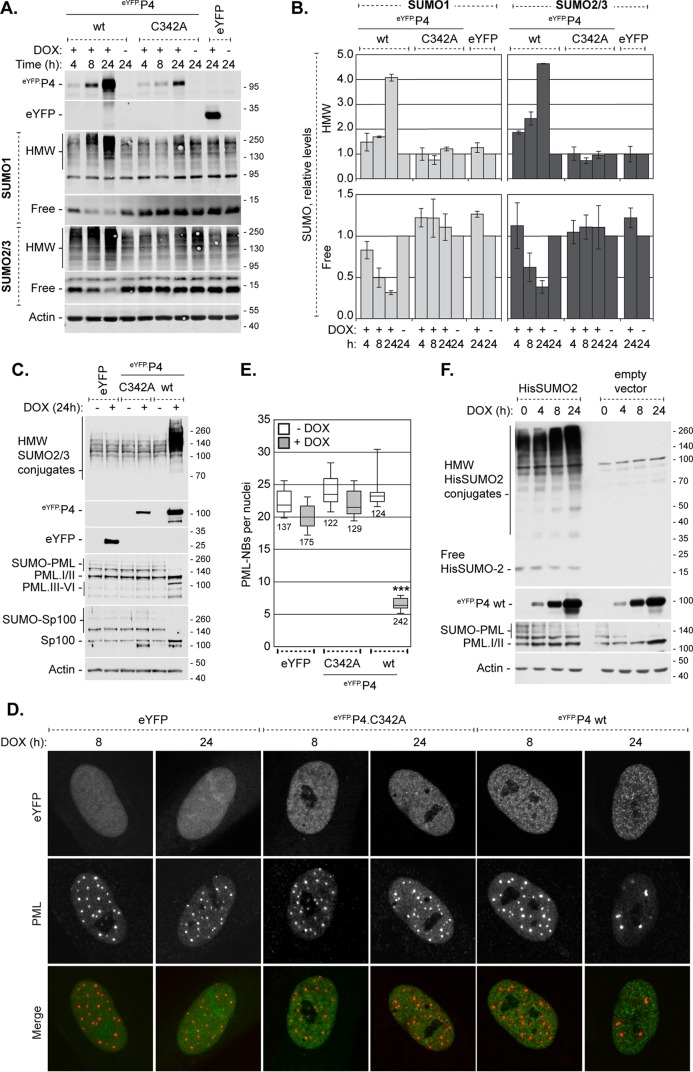
Overexpression of eYFP-PIAS4 disrupts SUMO pathway homeostasis. (A) Western blots show the levels of HMW-conjugated or free SUMO1 or -2/3 in transgenic HFt cells induced (+) to express eYFP or eYFP.PIAS4 wild-type (wt) or catalytically inactive mutant (C342A) proteins or not (−). Whole-cell lysates were collected at 4, 8, or 24 h postinduction. Membranes were probed for SUMO1 or -2/3, eYFP, or actin as a loading control. (B) Bar graphs show the average levels of HMW-conjugated or free SUMO1 or -2/3 in transgenic cells induced (+) or not (−) to express eYFP or eYFP.PIAS4 wild-type or catalytically inactive mutant proteins. The intensities of protein bands corresponding to SUMO1 or -2/3 quantitated from Western blots as in panel A were normalized to their respective loading control and expressed relative to the levels in uninduced cells (1.0). Means and SEM are shown (*n* = 2 [HMW] or *n* = 3 [free]). (C) Western blots show the SUMO modification of PML or Sp100 in transgenic cells induced (+) or not (−) to express eYFP or eYFP.PIAS4 wild-type or catalytically inactive mutant proteins for 24 h. Membranes were probed for SUMO2/3, eYFP, PML, Sp100, or actin as a loading control. (D) Confocal images show PML-NBs in transgenic cells induced to express eYFP or eYFP.PIAS4 wild-type or catalytically inactive mutant proteins. PML-NBs were identified by PML accumulation; PML was visualized by indirect immunofluorescence (red). (E) Box-and-whisker chart shows the number of PML-NBs per nuclei in transgenic cells induced (+DOX) for 24 h or not (−DOX) to express eYFP or eYFP.PIAS4 wild-type or catalytically inactive mutant proteins. Boxes, 25th to 75th percentile; black line, median number of PML-NBs per nucleus; whiskers, absolute range. The total number of cells analyzed per sample is indicated by each box from ≥10 fields of view per sample collected over two independent experiments. ***, *P* < 0.0001 (Mann-Whitney *U* test). (F) A Western blot shows the SUMO modification of PML in transgenic cells induced to express eYFP.P4 wt in the presence or absence of His-SUMO2. HFt cells stably transduced with eYFP.P4 expression vectors were transduced with a second lentiviral vector to constitutively express His-tagged SUMO2 or with an empty vector control. Expression of eYFP.P4 was induced for 0, 4, 8, or 24 h prior to the collection of whole-cell lysates. Membranes were probed for His, eYFP, PML, or actin as a loading control. Molecular mass markers are indicated, in kilodaltons.

EYFP.PIAS4 protein levels increased substantially with time, without considerable alteration to endogenous PIAS4 expression (Fig. 3C and D). As noted in other studies, however, high levels of ectopic PIAS4 had detrimental cellular effects (66, 68). Overexpression of eYFP.PIAS4 drastically affected SUMO signaling homeostasis, increasing HMW SUMO-conjugated protein levels at least 4-fold and decreasing free SUMO levels 5-fold following 24 h of induction (Fig. 4A and B). Consequently, dynamic SUMO modification was affected, which resulted in a loss of constitutive PML and Sp100 SUMOylation and disrupted PML-NBs (Fig. 4C to E), a phenotype similar to that in Ubc9-depleted cells (18). Consistently, the disruption of SUMO pathway homeostasis was attributed to PIAS4-mediated global depletion of free SUMO pools, as these effects did not occur when catalytically inactive PIAS4 was expressed or when His-tagged SUMO2 was expressed concomitantly with eYFP.PIAS4 to supplement the free SUMO2 pools (Fig. 4). To avoid gross disruption of SUMO pathway equilibrium, expression of the wild-type and mutant eYFP.PIAS4 proteins was typically induced for only 6 to 8 h, which did not substantially alter SUMO pathway homeostasis (Fig. 4A and B).

**FIG 5:**
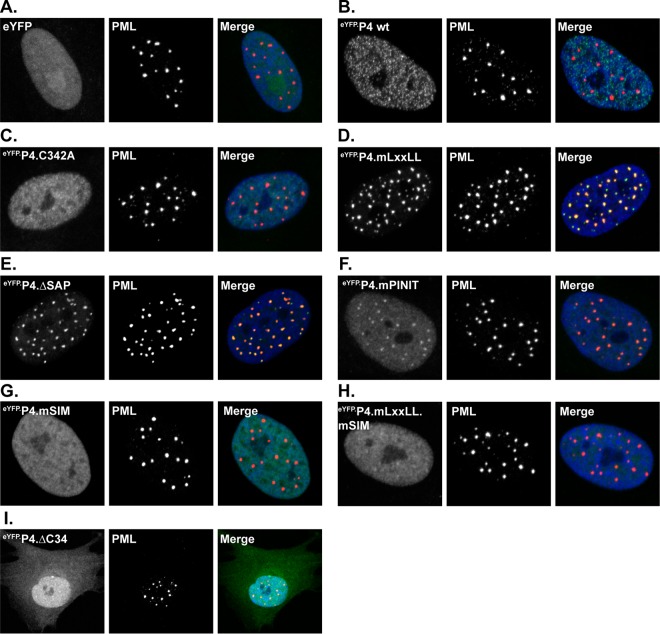
PIAS4 relocalizes to PML-NBs in a SIM- or ligase-dependent manner in the absence of SAP- or LxxLL-mediated interactions. Confocal images show the localization of eYFP or eYFP.PIAS4 wild-type or mutant proteins with respect to PML-NBs after 6 to 8 h of DOX induction. PML-NBs were identified by PML accumulation; PML was visualized by indirect immunofluorescence (red). Nuclei were visualized by DAPI (blue).

Amino-terminal fusion of eYFP did not adversely affect PIAS4 subnuclear localization (Fig. 5B). Inactivation of PIAS4 SUMO ligase activity (C342A), deletion of the SAP domain, or mutation of the LxxLL motif or SIM disrupted PIAS4 MAR localization (Fig. 5C to E and G). In contrast to the catalytically inactive or SIM mutant proteins, however, the ΔSAP and mLxxLL mutants localized to PML-NBs (Fig. 5D and E). Mutation of the PINIT motif resulted in an intermediate phenotype, with subpopulations of PIAS4 localized at PML-NBs or nuclear diffuse (Fig. 5F), suggesting that this motif stabilizes either localization. Deletion of the C-terminal AD (ΔC34) caused a nuclear diffuse and cytoplasmic localization, implicating this domain in nuclear retention or transport of PIAS4 (Fig. 5I).

These data demonstrate that all of the conserved domains of PIAS4 contribute to its characteristic MAR localization in HFt cells. In the absence of stable PIAS4 localization at MARs, the SIM or catalytic activity promotes localization at PML-NBs. The C-terminal AD of PIAS4 is required to stabilize PIAS4 localization at either MARs or PML-NBs and contributes to the predominantly nuclear localization of PIAS4.

### PIAS4 can colocalize with PML in ICP0-null mutant HSV-1 replication compartments in a SIM-dependent manner.

**FIG 6:**
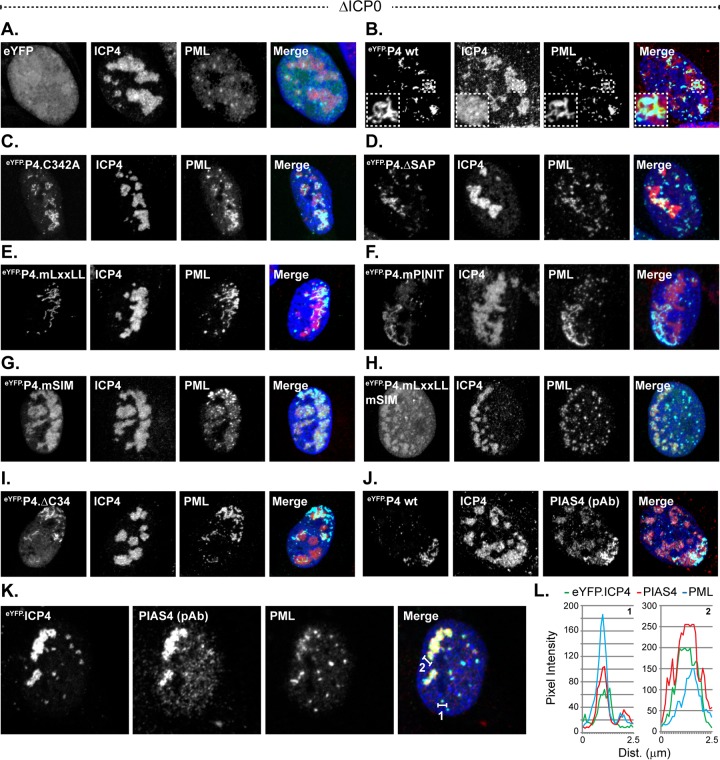
PIAS4 can associate with PML in ICP0-null mutant HSV-1 replication compartments in a SIM-dependent manner. (A to I) Confocal images show the nuclear localization of eYFP or eYFP.PIAS4 wild-type or mutant proteins (as indicated) with respect to ICP0-null mutant (ΔICP0) HSV-1 replication compartments, identified by ICP4 accumulation, or PML. Transgenic HFt cells were infected with 1 PFU of ICP0-null mutant HSV-1 per cell for 16 h prior to DOX induction of eYFP or eYFP.PIAS4 wild-type or mutant protein expression for 6 to 8 h. Localization of PML (cyan) and ICP4 (red) was visualized by indirect immunofluorescence. The inset (dashed box in panel B) highlights a region within a replication compartment where eYFP.P4 wt and PML colocalize in string-like structures (69). (J) Validation of PIAS4 polyclonal antibody (pAb; cyan) detection of eYFP.P4 wt within ICP0-null mutant HSV-1 replication compartments (ICP4; red). (K) Confocal images show the association of endogenous PIAS4 (red) with PML (cyan) in ICP0-null mutant HSV-1 replication compartments. (L) Emission spectra depict the pixel intensity and colocalization between eYFP.ICP4, endogenous PIAS4, and PML within a focus or region of an ICP0-null mutant HSV-1 replication compartment indicated by the corresponding numbered lines in panel K. Nuclei were visualized by DAPI (blue).

The subnuclear localization of mutant eYFP.PIAS4 proteins was initially evaluated during ICP0-null mutant HSV-1 infection to avoid potential ICP0-mediated disruption of relevant interactions. EYFP alone did not accumulate in replication compartments, while eYFP.PIAS4 did (Fig. 6A and B), demonstrating that fusion to eYFP did not adversely affect PIAS4 localization. However, endogenous PIAS4 tended to localize throughout individual replication compartments, whereas eYFP.PIAS4 tended to occupy subdomains within them (Fig. 6B and J). Notably, endogenous or exogenous PIAS4 could colocalize with PML, a known intrinsic antiviral factor (Fig. 6B, K, and L). The regions where eYFP.PIAS4 and PML colocalized were largely restrained to subdomains within individual replication compartments and often resembled string-like structures (Fig. 6B, inset) (69).

The mutant eYFP.PIAS4 proteins all accumulated in ICP0-null mutant HSV-1 replication compartments, although they had different localization phenotypes within them (Fig. 6C to I). As for wild-type eYFP.PIAS4, the ΔSAP, mLxxLL, mPINIT, ΔC34, and C342A mutant proteins could colocalize with PML (Fig. 4B to F and I). These regions of colocalization also tended to occupy subdomains within individual replication compartments, sometimes resembled string-like structures (69), and were not necessarily present within every replication compartment of any given cell. The colocalization of eYFP.PIAS4 mLxxLL, ΔSAP, or mPINIT mutants with PML in ICP0-null mutant HSV-1-infected cells was not unanticipated given that these mutants localized to PML-NBs in uninfected cells (Fig. 5D to F). Conversely, the eYFP.PIAS4 C342A and ΔC34 mutants only colocalized with PML in infected cells (compare Fig. 5C and I to 6C and I), which highlights a differential requirement for the ligase activity or the C-terminal AD of PIAS4 for colocalization with PML in uninfected cells.

Mutation of the PIAS4 SIM disrupted localization to PML-containing subdomains within replication compartments (Fig. 6G and H), consistent with the SIM-mediated localization of PIAS4 at PML-NBs in uninfected cells (Fig. 5G). In the absence of SIM-mediated interactions, PIAS4 still localized within replication compartments; however, it was homogenously distributed and tended to localize within all of the replication compartments of any given cell (Fig. 6G). Mutation of the SIM in conjunction with the LxxLL motif reduced PIAS4 accumulation within ICP0-null mutant HSV-1 replication compartments (Fig. 6H), indicating that the LxxLL motif also contributes to this localization phenotype.

These data demonstrate that PIAS4 can colocalize with PML within ICP0-null HSV-1 replication compartments, sometimes in string-like structures. The differential colocalization of PIAS4 with PML in cells that have been infected or not indicates that PIAS4 localization is differentially regulated during infection. While the SIM mediates colocalization with PML, such colocalization is not strictly required for PIAS4 accumulation in ICP0-null mutant HSV-1 replication compartments. This observation is consistent with multiple mechanisms to regulate PIAS4 subnuclear localization during infection.

### PIAS4 accumulation in wild-type HSV-1 replication compartments is largely mediated by the SAP domain or LxxLL motif.

**FIG 7:**
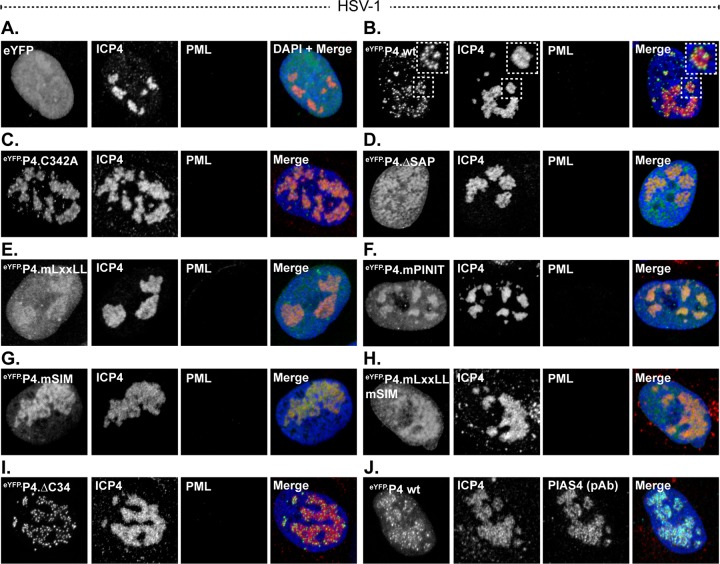
The SAP domain or LxxLL motif enhances PIAS4 accumulation in wild-type HSV-1 replication compartments. (A to I) Confocal images show the nuclear localization of eYFP or eYFP.PIAS4 wild-type or mutant proteins (as indicated) with respect to wild-type HSV-1 replication compartments, identified by ICP4 accumulation. Transgenic HFt cells were infected with 0.002 PFU of wild-type HSV-1 per cell for 16 h prior to DOX induction of eYFP or eYFP.PIAS4 wild-type or mutant protein expression for 6 to 8 h. ICP4 (red) and PML (cyan) were visualized by indirect immunofluorescence. The inset (dashed box in panel B) highlights the punctate localization of eYFP.P4 wt within a replication compartment. (J) Validation of PIAS4 pAb (cyan) detection of eYFP.P4 wt within wild-type HSV-1 replication compartments (ICP4; red). Nuclei were visualized by DAPI stain (blue).

The SIM-mediated colocalization of PIAS4 with PML was predominant, although not necessary for PIAS4 localization within replication compartments. The direct or indirect association of PIAS4 with PML could therefore conceal relevant interactions mediated by the other domains of PIAS4 that contribute to replication compartment localization. To address this possibility, the localization of wild-type and mutant eYFP.PIAS4 proteins was evaluated during wild-type HSV-1 infection, when ICP0 efficiently mediates PML degradation (70, 71). As for ICP0-null mutant HSV-1 infection, eYFP did not accumulate in wild-type HSV-1 replication compartments, whereas eYFP.PIAS4 did (Fig. 7A and B). The accumulation of eYFP.PIAS4 within wild-type HSV-1 replication compartments confirms that this localization is not disrupted by ICP0 and occurs independently of PML. EYFP-PIAS4 was distributed throughout wild-type HSV-1 replication compartments, similar to the distribution of endogenous PIAS4 (Fig. 7J). This distribution differed from that of eYFP.PIAS4 in ICP0-null mutant HSV-1 replication compartments, where it localized to subdomains within individual replication compartments (compare Fig. 6J and 7J). These data suggest that although fusion with eYFP does not adversely affect PIAS4 accumulation in replication compartments, it may stabilize the SIM-mediated association of PIAS4 with PML. SIM-dependent interactions, however, were not essential for PIAS4 localization in ICP0-null or wild-type HSV-1 replication compartments (Fig. 6G and 7G). Furthermore, it should be noted that eYFP fusion in itself did not promote association with PML (Fig. 5B).

The eYFP.PIAS4 C342A, mPINIT, and ΔC34 mutants localized to wild-type HSV-1 replication compartments (Fig. 7C, F, and I). The PINIT motif likely facilitates PIAS4 accumulation within replication compartments, as mutation of this motif increased the proportion of PIAS4 that was located outside of them (Fig. 7F). Mutation of the LxxLL motif or deletion of the SAP domain markedly reduced eYFP.PIAS4 accumulation within wild-type HSV-1 replication compartments, demonstrating that these domains largely facilitate or stabilize PIAS4 localization within them (Fig. 7D and E).

These data confirm that the localization of PIAS4 in HSV-1 replication compartments is not disrupted by ICP0. Whereas the PINIT motif promotes or stabilizes PIAS4 accumulation within replication compartments, the SAP domain or LxxLL motif considerably enhances it. However, as demonstrated during ICP0-null mutant HSV-1 infection, additional factors are sufficient for PIAS4 localization in replication compartments in the absence of a functional SAP domain or LxxLL motif.

### PIAS4 is recruited to domains that contain infecting HSV-1 genomes in a SIM-dependent manner that is disrupted by ICP0.

**FIG 8:**
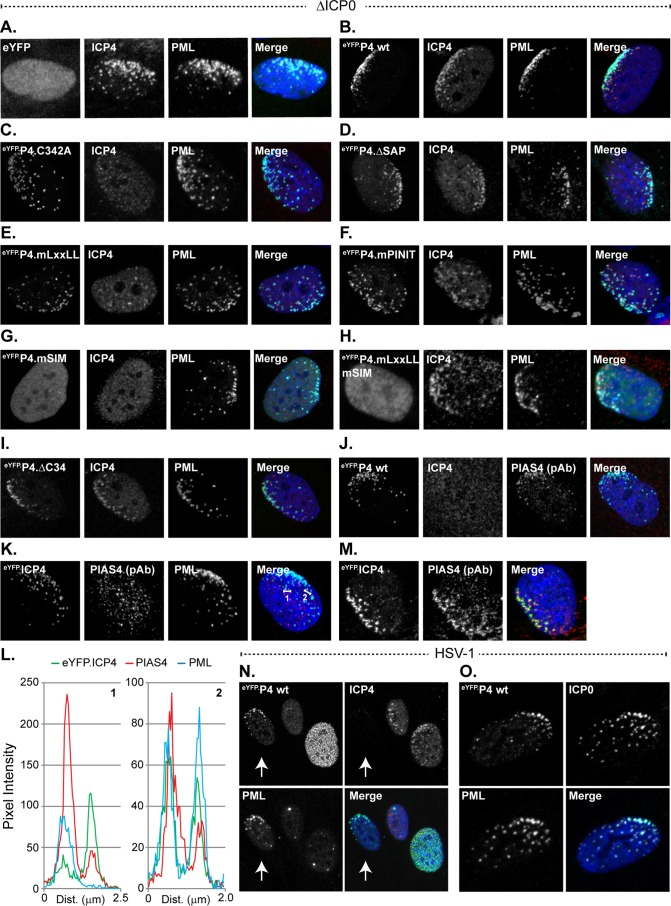
ICP0 disrupts PIAS4 SIM-dependent recruitment to nuclear domains associated with HSV-1 genome entry. (A to I) Confocal images show the nuclear localization of eYFP or eYFP.PIAS4 wild-type or mutant proteins (as indicated) with respect to infecting ICP0-null mutant (ΔICP0) HSV-1 genomes, visualized by the asymmetric nuclear edge localization of ICP4 or PML (6, 69). Transgenic HFt cells were infected with 1 PFU of ICP0-null mutant HSV-1 per cell for 16 h prior to DOX induction of eYFP or eYFP.PIAS4 wild-type or mutant protein expression for 6 to 8 h. ICP4 (red) and PML (cyan) were visualized by indirect immunofluorescence. (J) Validation of PIAS4 pAb (cyan) detection of eYFP.P4 wt at nuclear domains that contain infecting HSV-1 genomes. (K and M) Confocal images show the typical (K) or atypical (M) localization of endogenous PIAS4 (red) with respect to eYFP.ICP4 at a nuclear edge associated with ICP0-null mutant HSV-1 genome nuclear entry. (L) Emission spectra depict pixel intensity and colocalization between eYFP.ICP4 and PIAS4 in selected foci that correspond to the numbered lines in panel K. (N) Confocal images show the localization of eYFP.P4 wt with respect to PML or ICP4 in cells at the periphery of a developing wild-type HSV-1 plaque. Transgenic HFt cells were infected with 0.002 PFU of wild-type HSV-1 per cell for 16 h prior to induction of eYFP.P4 wt for 6 to 8 h. ICP4 (red) and PML (cyan) were visualized by indirect immunofluorescence. The arrow highlights the asymmetric redistribution of eYFP.P4 wt or PML to the nuclear edge prior to the detectable expression of ICP4. (O) Confocal images show the localization of eYFP.P4 wt with respect to ICP0 or PML prior to ICP0-mediated degradation of PML. Transgenic HFt cells were infected as for panel N. ICP0 (red) and PML (cyan) were visualized by indirect immunofluorescence. Nuclei were visualized by DAPI (blue).

**FIG 9:**
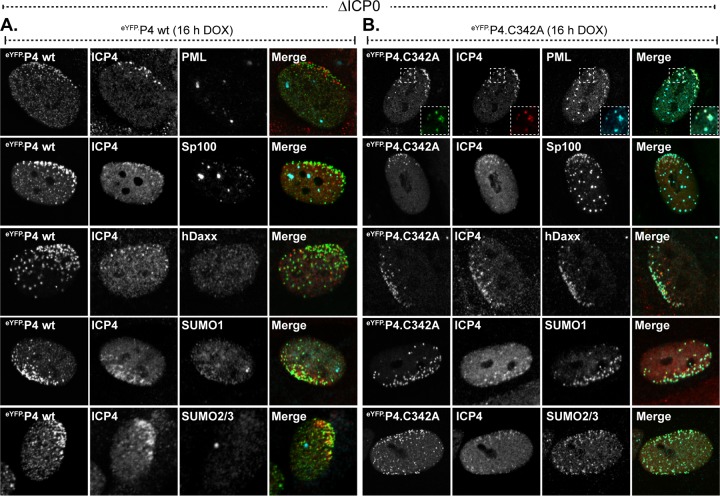
Overexpression of eYFP.PIAS4 disrupts the recruitment of PML-NB antiviral proteins to domains that contain infecting HSV-1 genomes in an SP-RING-dependent manner. (A and B) Confocal images show the recruitment of constituent PML-NB antiviral proteins to nuclear domains associated with HSV-1 genome entry in transgenic cells that express eYFP.PIAS4 wild-type (wt; A) or catalytically inactive mutant (C342A; B) proteins. Transgenic HFt cells were induced with DOX for 16 h before they were infected with 1 PFU of ICP0-null mutant (ΔICP0) HSV-1 per cell for 24 h. The nuclear domains that contain infecting HSV-1 genomes were identified by nuclear edge localization of ICP4. PML, Sp100, Daxx, SUMO1, or SUMO2/3 (cyan) and ICP4 (red) were visualized by indirect immunofluorescence.

During ICP0-null mutant HSV-1 infection, eYFP.PIAS4 also localized to nuclear domains that contained infecting viral genomes (Fig. 8B) (6, 8, 69). This localization was SIM dependent (Fig. 8C to I), consistent with the SIM-dependent recruitment of evaluated constituent PML-NB antiviral proteins (17). However, high levels of eYFP.PIAS4 expression disrupted the SIM-dependent recruitment of constituent PML-NB antiviral proteins without disrupting its own recruitment (Fig. 9). The global depletion of free SUMO pools as a consequence of high levels of eYFP.PIAS4 expression likely precluded the *de novo* SUMOylation events that mediate recruitment of constitutive PML-NB antiviral proteins (Fig. 4A and B and 9) (17, 18). In contrast, *de novo* SUMOylation, higher levels of free SUMO, or constituent PML-NB antiviral proteins were not required for the recruitment of eYFP-PIAS4 to domains that contained infecting HSV-1 genomes (Fig. 9A). Moreover, under conditions of depleted free SUMO pools, eYFP.PIAS4 was not sufficient to recruit constituent PML-NB antiviral proteins to nuclear domains that contained infecting HSV-1 genomes (Fig. 9).

EYFP.PIAS4 was recruited to domains that contained infecting HSV-1 genomes prior to the detectable expression of the IE protein ICP4 (Fig. 8J), indicating that recruitment occurs shortly after viral genomes enter the nucleus. Consistently, eYFP.PIAS4 was also recruited to nuclear domains that contained infecting wild-type HSV-1 genomes, although only prior to ICP0-mediated disruption of the intrinsic antiviral immune response as denoted by PML degradation (Fig. 8N and O). The redistribution of endogenous PIAS4 to sites associated with infecting HSV-1 genomes was not readily detected by immunofluorescence, although it could be observed on rare occasion (Fig. 8K and M). These data suggest that the recruitment of endogenous PIAS4 to domains that contain infecting HSV-1 genomes may be transient or unstable. Nonetheless, even in the absence of marked accumulation, endogenous PIAS4 could be detected in some foci that contained ICP4 and thus HSV-1 genomes by proxy (Fig. 8K and L).

These data demonstrate that PIAS4 is recruited through SIM-dependent mechanisms to nuclear domains associated with HSV-1 genome entry. The recruitment of constituent PML-NB antiviral proteins to these domains is not a prerequisite for PIAS4 recruitment, nor is PIAS4 sufficient to facilitate the recruitment of constituent PML-NB antiviral proteins when free SUMO pools are depleted. The recruitment of eYFP.PIAS4 under conditions of depleted free SUMO pools is consistent with a mechanism that is largely independent of *de novo* SUMOylation. Nonetheless, ICP0 is sufficient to disrupt the stable localization of PIAS4 at nuclear domains that contain infecting HSV-1 genomes.

### PIAS4 protein levels increase with the progression of HSV-1 infection.

**FIG 10:**
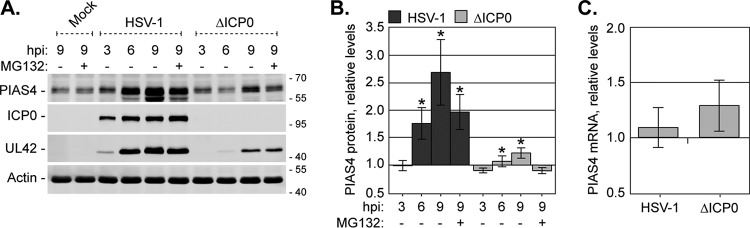
Endogenous PIAS4 expression increases during HSV-1 infection. (A) A Western blot shows PIAS4 levels during wild-type or ICP0-null mutant HSV-1 infection. HFt cells mock infected or infected with 10 PFU of wild-type or ICP0-null mutant (ΔICP0) HSV-1 per cell in the presence (+) or absence (−) of MG132 were harvested at 3, 6, or 9 hpi. Membranes were probed for PIAS4, either of the viral proteins ICP0 and UL42 to indicate infection progression, or actin as a loading control. Molecular mass markers are shown, in kilodaltons. (B) A bar graph shows the average levels of PIAS4 in wild-type or ICP0-null mutant HSV-1-infected cells. The intensities of PIAS4 protein bands quantitated from Western blots as in panel A were normalized to their respective loading controls and expressed relative to the normalized level in mock-infected cells at 9 hpi (1.0). Means and SEM are shown (*n* = 7). *, *P* < 0.05 (Student's two-tailed *t* test). (C) A bar graph depicts the average relative level of PIAS4 mRNA during wild-type or ICP0-null mutant HSV-1 infection. HFt cells were infected as described for panel A, and total RNA was isolated at 9 hpi. PIAS4 mRNA levels were determined using the TaqMan system of quantitative RT-PCR. Values normalized to GAPDH expression using the ΔΔ*C_T_* method are expressed relative to mock-infected cells (1.0). Means and SEM are shown (*n* ≥ 3).

The accumulation of endogenous PIAS4 in HSV-1 replication compartments increased in prominence as infection progressed, which could result from accumulation of PIAS4, an increase in PIAS4 protein expression, or both. The expression levels of PIAS4 were therefore evaluated in HFt cells infected with 10 PFU per cell of wild-type or ICP0-null mutant HSV-1. As infection progressed, endogenous PIAS4 protein levels increased (Fig. 10A and B). This increase was most prominent during wild-type infection, demonstrating that PIAS4 is not a target for ICP0-mediated degradation (Fig. 10A and B). Inhibition of the proteasome did not further enhance PIAS4 protein levels (Fig. 10A and B, +MG132), indicating that this increase was largely not achieved through changes to PIAS4 turnover rates. Although PIAS4 protein levels increased, PIAS4 mRNA levels remained largely unchanged (Fig. 10C), supporting the notion that PIAS4 does not correspond to an interferon-stimulated gene (ISG) (72). Together, these data suggest that the increase in PIAS4 protein levels is likely the result of changes to its translational or posttranslational regulation rather than changes to its transcription or protein turnover rates.

### PIAS4 restricts ICP0-null mutant HSV-1 replication through mechanisms complementary to PML-mediated restriction.

**FIG 11:**
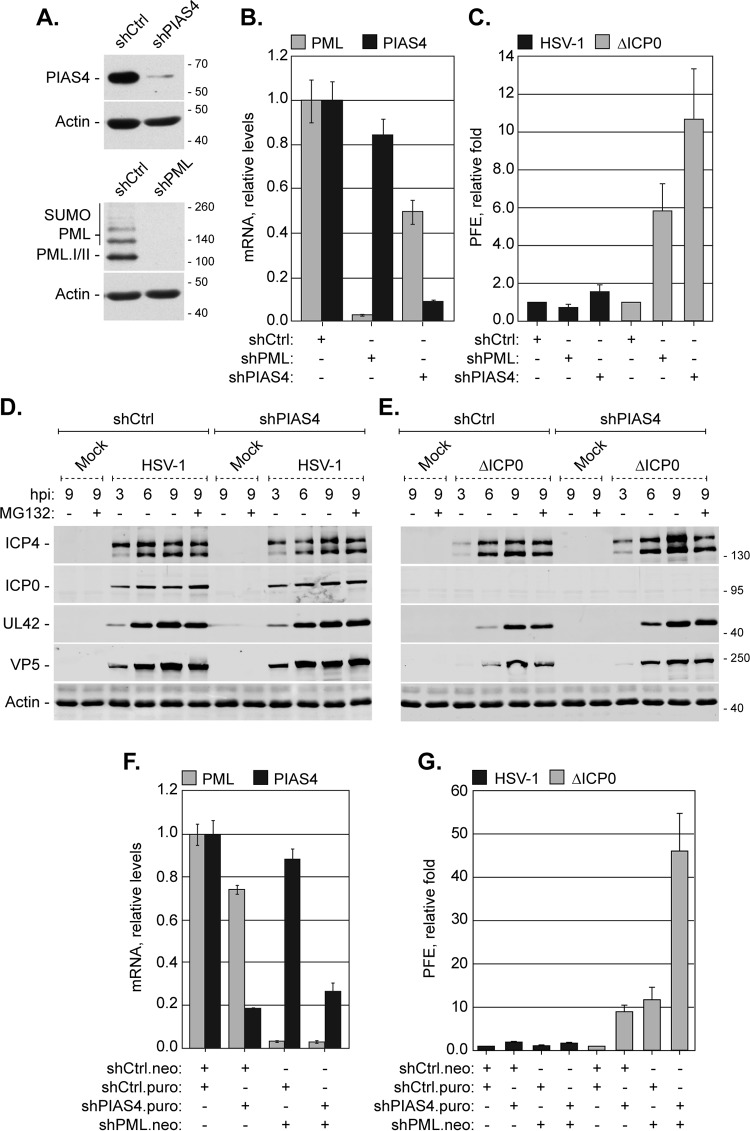
Endogenous PIAS4 contributes to the cellular restriction of ICP0-null mutant HSV-1. (A) Western blots show the levels of PIAS4 or PML in transgenic HFt cells that express short hairpin RNAs against PIAS4 (shPIAS4), PML (shPML), or a control sequence (shCtrl). Membranes were probed for PIAS4, PML, or actin as a loading control. (B) A bar graph shows the average relative levels of PIAS4 or PML mRNA in transgenic HFt cells that express shCtrl, shPIAS4, or shPML. PIAS4 or PML mRNA levels were determined using the TaqMan system of quantitative RT-PCR. Values normalized to 18S expression using the ΔΔ*C_T_* method are expressed relative to cells that expressed shCtrl (1.0). Values represent means and SD from three independent rounds of RT using mRNA isolated from one representative experiment. (C) A bar graph shows the average relative plaque formation efficiency (PFE) of wild-type or ICP0-null mutant (ΔICP0) HSV-1 in transgenic cells that express shCtrl, shPIAS4, or shPML, as indicated. The PFE for each strain in cells that express shPIAS4 or shPML was normalized to the respective PFE in cells that express shCtrl (1). Means and SD are shown (*n* ≥ 3). (D and E) Western blots show the levels of viral protein expression during wild-type or ICP0-null mutant (ΔICP0) HSV-1 infection of transgenic HFt cells that express shCtrl or shPIAS4. Transgenic cells mock infected or infected with 10 PFU of wild-type (D) or ICP0-null mutant (E) HSV-1 per cell in the presence (+) or absence (−) of MG132 were harvested at 3, 6, or 9 hpi. Membranes were probed for ICP4, ICP0, UL42, or VP5 to monitor the progression of infection or actin as a loading control. Molecular mass markers are shown, in kilodaltons. (F) A bar graph shows the average relative levels of PML or PIAS4 mRNA in transgenic cells that express shCtrl, shPIAS4, or shPML alone or in combination, as indicated. Analysis was performed as for panel B. Neo, neomycin resistance; Puro, puromycin resistance. (G) A bar graph shows the average relative PFE of wild-type or ICP0-null mutant HSV-1 in transgenic HFt cells that express shCtrl, shPIAS4, or shPML alone or in combination, as indicated. PFE analysis was done as described for panel C. Means and SD are shown (*n* ≥ 3).

PIAS4 expression increased during HSV-1 infection, and it localized to nuclear domains that contained viral genomes. Furthermore, PIAS4 colocalized with known intrinsic antiviral factors during ICP0-null mutant HSV-1 infection. To test the functional significance of PIAS4 during infection, replication of wild-type or ICP0-null mutant HSV-1 was evaluated in cells depleted of PIAS4 or of PML as a positive control (50). To this end, HFt cells were transduced with lentiviral vectors that expressed short hairpin RNAs (shRNAs) against PIAS4, PML, or a nontargeted scrambled control. Cells that expressed PML-specific shRNAs had a marked depletion in PML protein and mRNA levels, while cells that expressed PIAS4-specific shRNAs had considerable, although not complete, depletion of PIAS4 protein and mRNA levels (Fig. 11A and B). As PIAS4 is integral for cell division (45), the degree of PIAS4 depletion varied with each round of transduction, and after limited passaging, PIAS4 protein levels recovered to those in cells transduced with control shRNAs (data not shown). All functional experiments were therefore conducted over multiple rounds of independent transduction with minimal passaging following the isolation of stably transduced cells.

PML depletion did not affect the relative plaque forming efficiency (PFE) of wild-type HSV-1 and increased that of ICP0-null mutant HSV-1, as expected (Fig. 11C) (50). Depletion of PIAS4 did not noticeably affect the replication of wild-type HSV-1, demonstrating that PIAS4 is not essential for HSV-1 replication (Fig. 11C and D). PIAS4 depletion did, however, enhance the replication of ICP0-null mutant HSV-1 (Fig. 11C and E). In PIAS4-depleted cells, the PFE of ICP0-null mutant HSV-1 was enhanced 10-fold (Fig. 11C, ΔICP0). Furthermore, the expression and accumulation of viral proteins from all kinetic classes were increased in transgenic cells that expressed shPIAS4 relative to those in cells that expressed shCtrl (Fig. 11E). These data indicate that PIAS4 is an intrinsic antiviral factor that contributes to the cellular restriction of ICP0-null mutant HSV-1. However, expression of shRNAs against PIAS4 variably reduced PML expression (Fig. 11B and F and data not shown). These effects on PML were not a consequence of the specific sequence of the shRNA against PIAS4, as other evaluated PIAS4-specific shRNAs had similar effects (data not shown). Given the variable effects on PML expression when PIAS4 was depleted, it was possible that unintentional depletion of PML contributed to the relief of restriction of ICP0-null mutant HSV-1 replication in these cells. To address this possibility, replication of wild-type and ICP0-null mutant HSV-1 was evaluated in cells codepleted of PIAS4 and PML. HFt cells stably cotransduced with shRNAs against PML and PIAS4 had considerable depletion of both targets (Fig. 11F). Codepletion of PIAS4 and PML did not substantially alter the PFE of wild-type HSV-1, supporting the conclusions that PIAS4 is nonessential for HSV-1 replication and that any PIAS4-mediated antiviral activities are counteracted by ICP0 (Fig. 11G, HSV-1). Conversely, codepletion of PIAS4 and PML substantially enhanced the PFE of ICP0-null mutant HSV-1 to an even greater degree than depletion of either protein alone (Fig. 11G, ΔICP0). These data confirm that PIAS4 contributes to the restriction of ICP0-null mutant HSV-1 and demonstrate that the restrictive roles of PIAS4 are synergistic with those of PML. The SUMO E3 ligase PIAS4 is thus identified as an intrinsic antiviral factor.

## DISCUSSION

We show that PIAS4 is upregulated and localizes to nuclear domains that contain viral genomes during HSV-1 infection. The recruitment of PIAS4 to domains that contain infecting HSV-1 genomes is SIM dependent, whereas its accumulation in replication compartments is largely mediated by its SAP domain or LxxLL motif. However, in the absence of ICP0, the SAP domain or LxxLL motif is dispensable for PIAS4 localization in replication compartments, as the SIM mediates colocalization with PML within them. PIAS4 contributes to the restriction of ICP0-null mutant HSV-1 through mechanisms that are synergistic with those of PML. We thus identify PIAS4 as an intrinsic antiviral factor. This unique role for PIAS4 adds to the current understanding of PIAS proteins as immune regulators and warrants future investigation into their novel roles as intrinsic antiviral factors that restrict DNA virus infection.

Neither the STUbL-like activities of ICP0 nor the accumulation of HMW SUMO-conjugated proteins in the absence of ICP0 is sufficient to fully deplete the free SUMO pools during infection (Fig. 1A and B). Maintenance of free SUMO levels would uphold SUMO pathway integrity and permit the SUMO modification of specific targets throughout infection (28). SUMOylation could thus be utilized to influence the outcome of infection over the course of infection. Importantly, these data suggest that although ICP0 promotes the degradation of specific SUMOylated proteins, the SUMO moiety itself may not be degraded (18, 19, 28). Alternatively, SUMO protein expression may be upregulated (73). The almost full depletion (by 80 to 90%) of free SUMO following the overexpression of eYFP.PIAS4 or inhibition of the proteasome is in stark contrast to the levels of depletion during HSV-1 infection (20 to 30%) (Fig. 1B and 4B) and supports the concept that free SUMO pools are regulated during infection. Consistently, the broad changes in protein SUMOylation observed during HSV-1 infection would require a functional SUMOylation pathway and free SUMO (18, 28).

SUMO signaling is vital for the cellular restriction of ICP0-null mutant HSV-1. SUMO-SIM interactions regulate the individual recruitment of certain antiviral factors, including PIAS4 and constituent PML-NB antiviral proteins, to nuclear domains that contain infecting HSV-1 genomes (this study and reference 17). To our knowledge, the SIM-dependent recruitment of PIAS4 represents the first example of a nonconstituent PML-NB protein recruited in this manner. Disruption of *de novo* SUMOylation events, for example, through Ubc9 depletion, significantly impairs intrinsic immunity by hindering the recruitment of individual PML-NB antiviral proteins to domains that contain infecting HSV-1 genomes (17, 18). We now show that depletion of free SUMO pools also disrupts the recruitment of constituent PML-NB antiviral proteins to such domains, which further supports a requisite for new SUMOylation events in this process (Fig. 4B and 9). In contrast, the recruitment of (eYFP) PIAS4 to domains that contain infecting HSV-1 genomes is not adversely affected by the depletion of free SUMO pools (Fig. 9). This observation highlights a differential requirement for free SUMO in the mechanisms of recruitment for constituent PML-NB antiviral proteins and PIAS4. In the absence of relevant SIM-mediated interactions, other PIAS4 domains may be sufficient to mediate its recruitment. Alternatively, different SIM-dependent mechanisms of recruitment may exist, for example, through interactions with existing versus newly SUMOylated targets. Even so, ICP0 is sufficient to disrupt the stable localization of PIAS4 at nuclear domains associated with HSV-1 genome entry.

In the absence of ICP0, it is tempting to relate the localization of SUMO2/3 and PIAS4 in viral replication compartments to the accumulation of HMW SUMO-conjugated proteins. Of the relatively few SUMO E3 ligases identified, PIAS4 is the only one evaluated that extensively localizes within HSV-1 replication compartments (Fig. 2; also evaluated were RanBP2 and KAP1 [data not shown]). However, how PIAS4 catalytic activity is regulated, or if PIAS4 even mediates SUMOylation events during infection, is not yet known. It will be interesting to determine if the antiviral activities of PIAS4 are indeed accomplished through its catalytic activity, as SUMO ligation is dispensable for many of its characterized functions (34, 66–68, 74–84). The localization of PIAS4 at nuclear domains that contain HSV-1 DNA occurs independently of its catalytic activity, and its roles at these sites may also be independent of SUMO ligation. During ICP0-null mutant HSV-1 infection, the accumulation of HMW SUMO-conjugated proteins was not substantially altered in PIAS4-depleted cells relative to that in their transduction controls (data not shown). This observation could argue that PIAS4 is not the sole or primary SUMO E3 ligase to catalyze these SUMOylation events. However, the levels of PIAS4 depletion may not have been to a degree that would detectably alter the accumulation of HMW SUMO-conjugated proteins within the population of depleted cells. Further investigation into the regulation of PIAS4 catalytic activity and the identification of potential PIAS4 substrates in infected cells is warranted.

Stabilization of certain domain-mediated interactions over others could provide a rapid and dynamic means to alter PIAS4 subnuclear localization or binding partners to quickly respond to changing nuclear conditions. In noninfected HFt cells, the interactions that mediate PIAS4 MAR association are favored over those that mediate localization at other nuclear domains, such as PML-NBs (Fig. 5). During infection with ICP0-null mutant HSV-1, PIAS4 SIM-mediated direct or indirect interactions with PML predominate (Fig. 6B and G and 8B and G), suggesting that infection enhances PIAS4 SIM-mediated interactions over those mediated by its other domains. Whereas the PIAS4 SIM-dependent recruitment to nuclear domains that contain infecting HSV-1 genomes is efficiently disrupted by ICP0, its SAP domain- or LxxLL motif-mediated accumulation in replication compartments is not (Fig. 2D and 8B and N). The ICP0-sensitive recruitment of PIAS4 to domains that contain infecting HSV-1 genomes suggests that this is where PIAS4 exerts its antiviral activities. Consequently, the ICP0-mediated degradation or dispersal of relevant SUMOylated proteins to prevent PIAS4 recruitment could efficiently counteract PIAS4 antiviral activities (18). However, the potential for PIAS4-mediated antiviral roles within replication compartments cannot be ruled out. Multiple mechanisms to recruit PIAS4 into replication compartments may represent a cellular antiviral “failsafe” or could indicate that PIAS4 has multiple roles during infection. An overarching theme in the characterized functions of PIAS4 is the availability of cellular cofactors or subnuclear localization to dictate PIAS4 activity in any given role, which supports the plausibility of the latter scenario. PIAS4 differentially localizes within replication compartments in the presence or absence of ICP0 (compare Fig. 6B and J to 7B and J), which corroborates the concept that PIAS4 has different interaction partners under these different infection conditions. However, PIAS4 is only restrictive in the absence of ICP0 (Fig. 11C and G). Thus, ICP0 could also counteract the antiviral activities of PIAS4 within replication compartments by disrupting relevant PIAS4 interactions or localization within them. Identification of PIAS4 interaction partners during wild-type or ICP0-null mutant HSV-1 infection will aid in understanding how PIAS4 contributes to intrinsic antiviral immunity and how its antiviral effects are counteracted by ICP0.

The increase in PIAS4 protein expression during infection is most likely achieved through changes in its translational or posttranslational regulation (Fig. 10). The 3′ untranslated region of PIAS4 mRNA has mir-29 and let-7 microRNA family complementary sites (TargetScanHuman release 7.0 [85]). Downregulation of mir-29 or let-7 during infection provides one means whereby PIAS4 translation could be altered to increase protein expression (73, 86–88). As infection progresses, ICP0 accumulates in the cytoplasm, where it antagonizes IRF3-mediated transactivation (89, 90). The upregulation of PIAS4 could aid in the repression of innate immunity through PIAS4-mediated antagonism of STAT and NF-κB transactivation (34, 43). Moreover, PIAS4 also directly binds to and represses IRF3, which could provide an added level of IRF3 suppression during infection (37, 78).

PIAS proteins were initially characterized as negative regulators of innate immunity. Depletion of PIAS4 would thus be expected to increase IFN and ISG production and further restrict replication of IFN-sensitive viruses. Partially consistent with this concept, type I IFN is upregulated in PIAS4^−/−^ mouse embryonic fibroblasts (MEFs) (78), while STAT1 or NF-κB gene activation is not detectably altered (43). Even with the variable effects of PIAS4 knockdown on innate immune signaling, Sendai virus replication was further restricted in PIAS4^−/−^ MEFs (78). In contrast, depletion of PIAS4 in HFt cells enhanced the replication of ICP0-null mutant HSV-1. This observation was somewhat unexpected, as ICP0-null mutant HSV-1 is hypersensitive to repression by innate immunity (91, 92). However, the assay used to evaluate the PFE of HSV-1 in PIAS4 depleted cells is likely biased toward intrinsic, rather than innate, immunity. As the PFE assay largely measures the productivity of the initial infection event, the IFN response is likely too slow to impede viral replication given that signaling pathway activation and ISG synthesis are required to mount a successful defense. Therefore, intrinsic immunity-mediated repression likely largely underlies any PFE defects. Consistent with this proposal, the PFE of ICP0-null mutant HSV-1 is not enhanced in cells with impaired innate immunity, achieved through depletion of STAT1 or IRF3, while it is enhanced in cells with impaired intrinsic immunity (10, 18, 50, 93, 94). Our identification of PIAS4 as a component of the intrinsic antiviral immune response suggests that PIAS proteins may sit at a crux of regulation to relate intrinsic and innate immunity. It is conceivable that a factor that positively regulates intrinsic immunity would downregulate innate immunity to limit situations in which a positive feedback loop enhances the overall immune response such that it cannot be curtailed following successful clearance or repression of the invading pathogen. We have also recently identified PIAS1 as an intrinsic antiviral factor (unpublished data), suggesting that the complementary and cooperative roles of PIAS proteins in the repression of innate immunity may be mirrored in the activation of intrinsic immunity. Further characterization of the roles of PIAS4 in immune regulation will identify how it balances regulation of the innate and intrinsic immune responses and highlight points that may be manipulated in the future to facilitate cellular clearance or silencing of viral pathogens.
